# Self-Damage Caused by Dysregulation of the Complement Alternative Pathway: Relevance of the Factor H Protein Family

**DOI:** 10.3389/fimmu.2018.01607

**Published:** 2018-07-12

**Authors:** Pilar Sánchez-Corral, Richard B. Pouw, Margarita López-Trascasa, Mihály Józsi

**Affiliations:** ^1^Complement Research Group, Hospital La Paz Institute for Health Research (IdiPAZ), La Paz University Hospital, Center for Biomedical Network Research on Rare Diseases (CIBERER), Madrid, Spain; ^2^Department of Pharmaceutical Sciences, University of Basel, Basel, Switzerland; ^3^Department of Medicine, Universidad Autónoma de Madrid, Madrid, Spain; ^4^Complement Research Group, Department of Immunology, ELTE Eötvös Loránd University, Budapest, Hungary; ^5^MTA-SE Research Group of Immunology and Hematology, Hungarian Academy of Sciences and Semmelweis University, Budapest, Hungary

**Keywords:** age-related macular degeneration, atypical hemolytic uremic syndrome, C3 glomerulopathy, complement activation, complement de-regulation, factor H, factor H-related protein, opsonization

## Abstract

The alternative pathway is a continuously active surveillance arm of the complement system, and it can also enhance complement activation initiated by the classical and the lectin pathways. Various membrane-bound and plasma regulatory proteins control the activation of the potentially deleterious complement system. Among the regulators, the plasma glycoprotein factor H (FH) is the main inhibitor of the alternative pathway and its powerful amplification loop. FH belongs to a protein family that also includes FH-like protein 1 and five factor H-related (FHR-1 to FHR-5) proteins. Genetic variants and abnormal rearrangements involving the FH protein family have been linked to numerous systemic and organ-specific diseases, including age-related macular degeneration, and the renal pathologies atypical hemolytic uremic syndrome, C3 glomerulopathies, and IgA nephropathy. This review covers the known and recently emerged ligands and interactions of the human FH family proteins associated with disease and discuss the very recent experimental data that suggest FH-antagonistic and complement-activating functions for the FHR proteins.

## Introduction

While initially only regarded as a supporting factor for the effectivity of immunoglobulins, the complement system is nowadays widely recognized as a crucial part of the innate immune system involved in many different processes (1). In addition to acting as a first line of defense by directly targeting and killing invading pathogens, with or without the help of immunoglobulins, its role in inflammation, immune cell recruitment, and clearance of immune complexes, apoptotic cells, and necrotic cells places complement at the center of the human immune system. The relevant role of complement is corroborated by the variety of pathological situations associated with complement deficiency or dysfunction.

**Figure 1:**
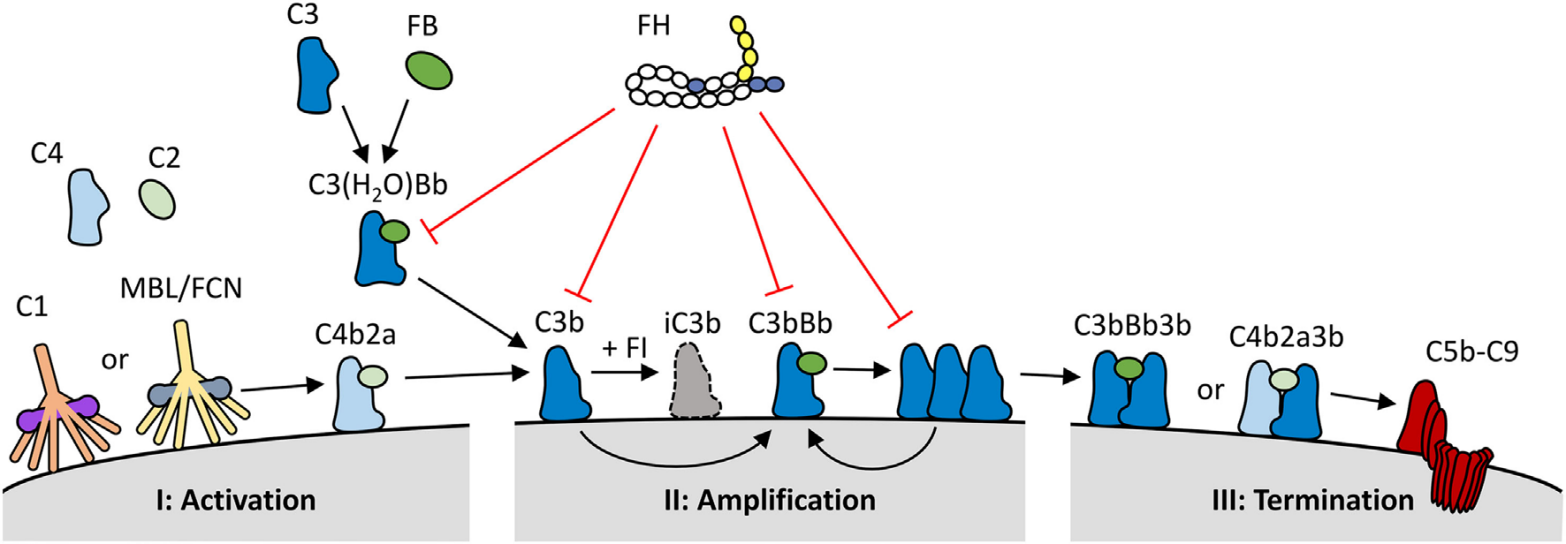
Overview of the role of factor H (FH) within the complement system. (I) The complement system is activated *via* binding of C1q (classical pathway), or mannan binding lectin/ficolins (MBL/FCN) (lectin pathway) in complex with serine proteases to specific molecules, or through the spontaneous activation of C3 into C3(H_2_O) (alternative pathway). Upon activation, the three pathways form C3 convertases (C4b2a or C3(H_2_O)Bb) resulting in the generation and deposition of C3b on the activating surface. (II) C3b forms new C3 convertase molecules (C3bBb) that enhance C3b deposition and amplify complement activation. (III) C3b can also bind to C3 convertases to generate C5 convertases (C4b2a3b or C3bBb3b); this process initiates the terminal pathway of complement activation, and the formation of the lytic C5b–C9 complex. FH keeps the spontaneous activation of C3 under control, and it also inhibits the complement system at both the activation and amplification stages. FH binds to deposited C3b and C3bBb complexes on human cell surfaces and inhibits further activation by three mechanisms: it competes with factor B (FB) for C3b binding and C3bBb generation; it increases the decay of C3bBb complexes, and it acts as a cofactor for factor I (FI), which in turn cleaves C3b into inactive C3b (iC3b).

Three complement activation pathways have been defined, each comprised of various proteins forming an intricate cascade of activation events (Figure 1). Both the classical and the lectin pathways are initiated when pattern recognition molecules (PRMs) that are complexed with zymogens of serine proteases, bind to their ligand. The classical pathway is activated by the binding of the C1 complex to immunoglobulins and pentraxins, while the lectin pathway uses various PRMs, including mannose-binding lectin and ficolins, which bind to specific carbohydrate moieties. These ligands are normally not present on healthy human cells. In contrast, the alternative activation pathway is initiated through the constitutive low rate hydrolysis of the internal thioester bond of C3, allowing binding of various activating complement proteins. All three pathways lead to the cleavage of C3 into C3a and C3b. C3b contains a highly reactive thioester group that is exposed upon C3 cleavage, resulting in the deposition of C3b onto virtually any molecule or cell surface in close proximity. When left unchecked, C3b on its own will again initiate the alternative pathway. As both the classical and the lectin pathway will also activate the alternative pathway once C3b is formed, thus enhancing complement activation, the alternative pathway has a pivotal role as an amplification loop within the complement system. Up to 80% of total complement activation has been ascribed to this amplification loop (2). Due to the spontaneous nature of the alternative pathway, it must be tightly controlled to prevent unwarranted and dangerous complement activation.

**Figure 2:**
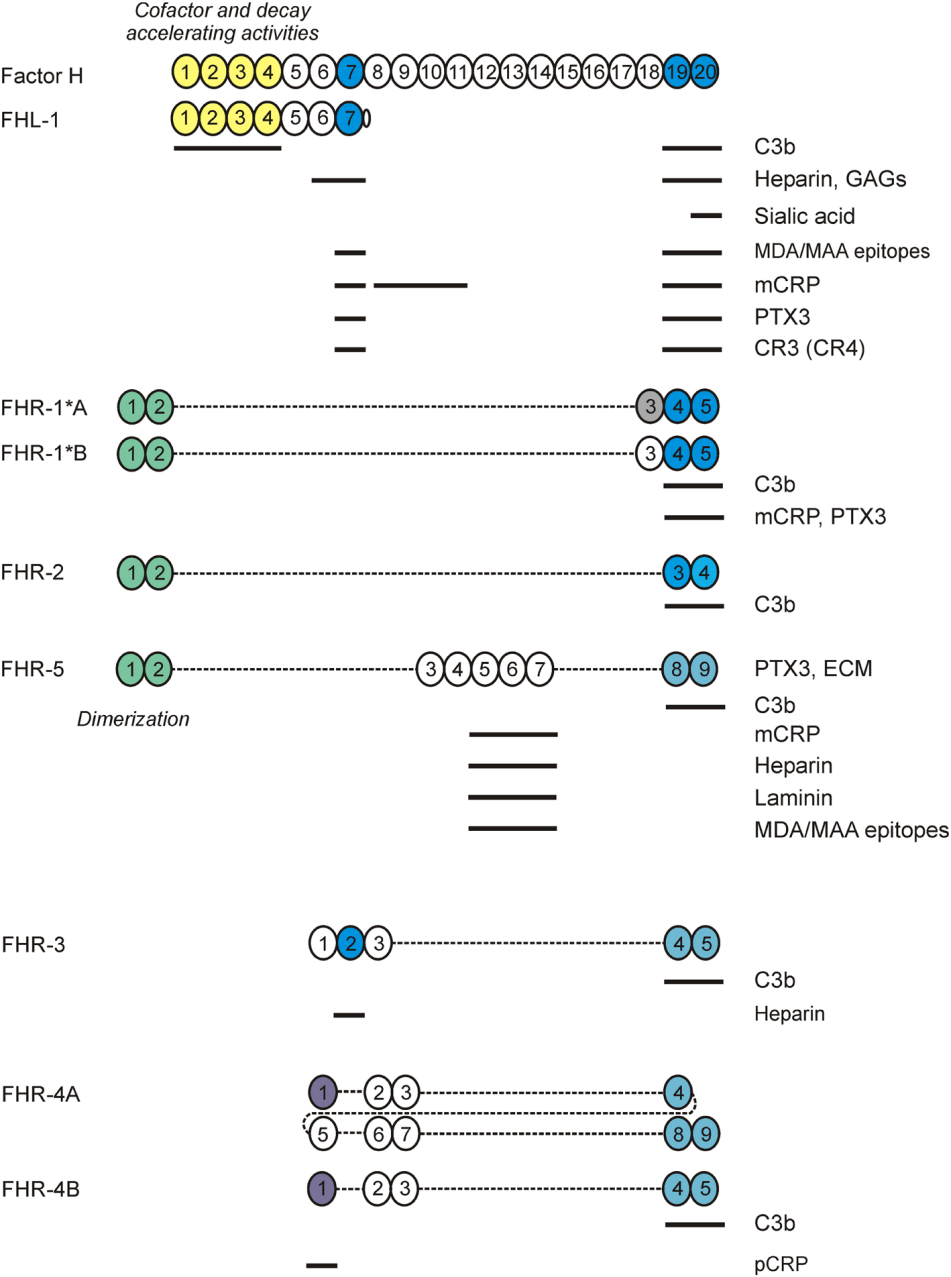
Factor H (FH) family proteins and their ligands. The schematic complement control protein (CCP) domain structure of FH, FHL-1, and the FHR proteins is shown, with CCPs aligned vertically to the homologous domains in FH. The N-terminal CCPs 1–4 of FH and FHL-1 mediate the complement regulatory functions of these proteins (shown in yellow). CCPs 7 and 19–20 (shown in blue) harbor the main ligand- and host surface-recognition sites; selected ligand binding sites are indicated by horizontal lines. The CCPs 1–2 of factor H related protein 1 (FHR)-1, FHR-2, and FHR-5 are closely related to each other and mediate dimerization of these FHRs. The CCPs in FHRs with high sequence identity to the homologous FH domains are indicated by identical/similar colors.

Complement regulation takes places both on the human cell surface and in the fluid phase. Several regulators, like most complement components, are found in the circulation. In addition, human cells express a wide array of membrane-bound complement regulators that control the system at various steps. Especially due to the activating proteins of the alternative pathway, regulation in the fluid phase is crucial, as unchecked, spontaneous C3 activation would lead to complete consumption of C3 and loss of complement activity. The 155-kDa glycoprotein complement factor H (FH) is the major regulator of the alternative pathway, inhibiting C3 activation both in the fluid phase as well as on human cell surfaces (Figure 1) (3). Similar to other complement regulators encoded in the regulators of complement activation (RCA) gene cluster, FH is composed of complement control protein (CCP) domains, often also referred to as short consensus repeat domains. FH is composed of 20 CCPs (Figure 2) (4). The first four N-terminal domains contain the complement inhibiting activity, such as decay accelerating activity and co-factor activity (5). The two most C-terminal CCP domains (19 and 20), together with a region located in CCPs 6–8, are crucial for binding of FH to surfaces, such as human cell membranes, as well as for mediating binding to several host and non-host ligands (discussed below) (6, 7). FH is highly abundant in plasma, with circulating levels of 233–400 µg/mL on average, although it has to be noted that some of the assays used might detect other FH family members as well (8–11).

Factor H-like protein 1 (FHL-1) is a splice variant derived from the *CFH* gene. Serum levels of FHL-1 are estimated to be 10–50 µg/mL (12, 13). FHL-1 is identical to the first seven CCP domains of FH, with an unique, four amino acid long C-terminus (14, 15). Thus, FHL-1 shares the C3b binding and regulatory domains CCPs 1–4 with FH and, like FH, it has complement inhibiting activity (16). Likewise, due to the shared CCPs 6–7 domains, FHL-1 and FH bind some common ligands, such as heparin, the pentraxins C-reactive protein (CRP) and pentraxin 3 (PTX3), and malondialdehyde epitopes (Figure 2). However, there are also differences in ligand interactions between FHL-1 and FH, not only because of the extra domains in FH but also due to the difference in their conformation and the unique SFTL tail at the C-terminus of FHL-1. For example, it was recently reported that the SFTL tail increases the interaction of FHL-1 with CRP and PTX3 (17).

Next to FH and FHL-1, humans (and several non-human species; not discussed here) possess FH-related (FHR) proteins, homologous to FH. They are encoded separately, with their genes (*CFHR1* to *CFHR5*) lying in tandem next to *CFH* at 1q31.3. The *CFHR* genes originate from *CFH* through gene duplication events (18). The *CFH–CFHRs* loci contain several segmental duplications, making them prone to genetic structural rearrangements due to nonallelic homologous recombination (NAHR) events. This has led to copy number polymorphisms (CNPs), with the very common 86.3-kb deletion (CNP147) that results in loss of *CFHR3–CFHR1* (Δ*CFHR3–CFHR1*), and the very rare 122-kb deletion (CNP148) resulting in loss of *CFHR1–CFHR4* (Δ*CFHR1–CFHR4*) (19). Like FH, the FHRs are entirely composed of CCP domains (Figure 2), which display high sequence similarity with CCP domains of FH known to be involved in ligand and surface binding. Remarkably, none of the human FHR proteins possess CCP domains homologous to FH CCPs 1–4. Thus, based on their primary structure, FHR proteins are not expected to have any direct complement inhibiting activity similar to FH. Nonetheless, several reports have observed direct complement inhibitory activity for some of the FHRs, albeit often weak compared to FH (20–24). However, other studies have not found such activity for FHRs, questioning whether this is truly the physiological role of the FHR proteins (25–30). Instead, the FHRs are currently hypothesized to have an antagonistic function over FH, competing with binding to FH ligands and cell surfaces. By lacking direct complement inhibiting activity, binding of FHRs instead of FH would allow complement activation to proceed (31). This process has also been termed complement de-regulation. Indeed, binding to various (FH) ligands has been reported for all FHRs, which will be discussed below. In addition, some FHRs were reported to promote alternative pathway activation by binding C3b and serving as a platform for the assembly of the C3 convertase (27, 32, 33). Recent characterization of some of the mouse FHRs supports a role of these proteins as positive regulators in the modulation of complement activation (34, 35).

In this review, we outline and provide an update on the recent developments regarding the FH protein family. New insights regarding circulating levels of FHRs, ligand binding, and disease associations allow re-assessing the role of FHRs in the complement system. Together, these results shed light on the balance of the FH–FHRs axis, and the role of FHRs in non-pathological and pathological conditions.

## Quantitation of FHR Proteins

Factor H, FHL-1, and the FHR proteins are mainly synthesized by hepatocytes, but synthesis by other cells and tissues has also been reported, particularly for FH and FHL-1 (36–38). FH production has been detected in endothelial cells, platelets, mesangial cells, keratinocytes, fibroblasts, retinal pigment epithelial cells, monocytes, and dendritic cells, among others (39–46). On the other hand, little information on the extrahepatic expression of the FHR proteins is available. Both *CFHR3* mRNA and FHR-3 protein have been identified in retinal macrophages, while no FHR-3 expression was found in other retinal cell types (47). Extrahepatic synthesis of FH/FHRs most likely contributes to an efficient control of complement activation locally, but a relevant contribution to the plasma levels of these proteins is unlikely, considering the relative low expression compared to the hepatic source.

Accurate quantification of the FHR proteins has been a great challenge since their discovery. Due to the high sequence similarity among FH and FHR proteins, it has proven to be very difficult to obtain specific reagents for each of the FHR proteins. For some time, only concentration estimates were available for most of the FHR proteins (21, 48). However, with recently renewed and successful efforts in generating highly specific antibodies, specific immunoassays for each of the FHR proteins are now becoming available, although some discrepancy about their actual physiological levels still remains (Table 1).

**Table 1:** Reported serum levels of the factor H-related (FHR) proteins.

Protein measured	Gene copies	Levels (μg/mL)		*N*	Reference
Factor H (FH)	–	400	SD = 62	1,004	(8)
	–	319.9	SD = 71.4	358	(9)
	–	233.24	SD = 56.65	63	(10)
	–	232.7	SD = 74.5	1,514	(11)
FHL-1	–	47	SD = 11.3^a^	2	(12)
Total factor H-related protein 1 (FHR-1)	1*CFHR1	61	SD = 31	24	(54)
	2*CFHR1	122	SD = 26	44	(54)
	Not defined	94	IQR = 70.5–119.6	158	(55)
	Not defined	1.63	SD = 0.04	344	(66)
FHR-1 homodimers	1*CFHR1	4.88	SD = 1.33	36	(53)
	2*CFHR1	14.64	SD = 3.04	77	(53)
FHR-1/2 heterodimers	1*CFHR1	5.01	SD = 1.49	36	(53)
	2*CFHR1	5.84	SD = 2.41	77	(53)
FHR-2 homodimers	0*CFHR1	3.1		Pool of four donors	(53)
	1*CFHR1	0.85	SD = 0.41	36	(53)
	2*CFHR1	0.65	SD = 0.41	77	(53)
Total FHR-2	Not defined	3.64	SD = 1.2	344	(66)
FHR-3	1*CFHR3	0.38	SD = 0.23	26	(58)
	2*CFHR3	0.83	SD = 0.48	69	(58)
	2*CFHR3*A	0.55	SD = 0.15	16	(60)
	2*CFHR3*B	0.82	SD = 0.08	4	(60)
	Not defined	1.06	SD = 0.53	21	(47)
	Not defined	0.020	SD = 0.001	344	(66)
FHR-4A	–	25.4	Range = 6.5–53.9	11	(27)
	–	2.42	SD = 0.18	344	(66)
	–	2.55	SD = 1.46	129	(63)
FHR-4B	–	Not detected		–	(63)
FHR-5 homodimers	–	5.5	Range = 3.4–10.1	13	(65)
	–	5.49	SD = 1.55	344	(66)
	–	2.46	IQR = 1.79–3.67	158	(55)
	–	1.66	SD = 0.43	115	(53)

*^a^FHL-1 levels were determined indirectly, by subtracting the values of FH measurements from those of FH + FHL-1 measurements. N: number of samples; SD: standard deviation; IQR: interquartile range*.

### Factor H-Related Protein 1 (FHR-1)

Factor H-related protein 1 is composed of five CCP domains, and circulates in two forms (37 and 42 kDa), with either one or two *N*-linked carbohydrate moieties (30, 49, 50). Two genetic variants of FHR-1 have been described, FHR-1*A and FHR-1*B, the difference being three amino acids in CCP3 (51). FHR-1*B CCP3 is identical to FH CCP18, whereas FHR-1*A CCP3 shares 95% sequence identity with FH CCP18. FHR-1 CCPs 4 and 5 share high sequence identity (100 and 97%) with FH CCPs 19 and 20, respectively. FHR-1 has a dimerization motif located in CCPs 1–2 that are highly similar (>85% sequence identity) to CCPs 1–2 of FHR-2 and FHR-5, and allow the formation of FHR-1 homodimers and heterodimers with FHR-2 (26, 52, 53). While identified *in vitro*, the existence of FHR-1/FHR-5 heterodimers *in vivo* is still controversial (26, 52, 53). Similarly, FHR-1 quantification also remains controversial. In 2017, several groups determined FHR-1 levels. Tortajada et al. reported an average of 122 µg/mL in 44 healthy controls with two copies of *CFHR1*, and an overall average of 90.4 µg/mL in 76 controls (including eight homozygous Δ*CFHR3*–*CFHR1* carriers and 24 heterozygous Δ*CFHR3*–*CFHR1* carriers) (54). Using the same immunoassay, Medjeral-Thomas et al. reported 94.4 µg/mL FHR-1 in 158 controls (of whom 133 were genotyped: 3 Δ*CFHR3*–*CFHR1* homozygous, 45 Δ*CFHR3*–*CFHR1* heterozygous, and 85 without Δ*CFHR3*–*CFHR1*) (55). Of note, the immunoassay described by Tortajada et al. does not distinguish between FHR-1 homodimers or heterodimers. In contrast, using immunoassays specific for FHR-1 homodimers and FHR-1/-2 heterodimers, van Beek et al. reported ~10-fold lower levels (averages of 11.33 and 5.48 µg/mL, respectively), in 115 healthy donors (2 homozygous Δ*CFHR3*–*CFHR1*, 36 heterozygous Δ*CFHR3*–*CFHR1* carriers, and 77 without Δ*CFHR3*–*CFHR1*) (53).

### Factor H-Related Protein 2

Factor H-related protein 2 is the smallest FHR protein, composed of four CCP domains (56). FHR-2 circulates either non-glycosylated (24 kDa) or with one *N*-linked carbohydrate moiety in CCP2 (29 kDa). FHR-2 CCP1 and CCP2 are nearly identical to FHR-1 CCP1 and CCP2 (100 and 98%), respectively, including all residues comprising the dimerization motif (26). Similar to the proposed FHR-1/FHR-5 dimers, FHR-2/FHR-5 dimers remain to be identified *in vivo*, while FHR-2 homodimers and FHR-1/FHR-2 heterodimers have been confirmed (52, 53). FHR-2 homodimer levels have been shown to be around 3 µg/mL; with these relatively low levels, FHR-2 seems to be the limiting factor in the formation of FHR-1/FHR-2 heterodimers and, indeed, most FHR-2 is found dimerized with FHR-1 (53).

### Factor H-Related Protein 3

Factor H-related protein 3 is composed of five CCP domains, of which CCP1 and CCP2 have high sequence similarity with FH CCP6 and CCP7 (94 and 86%), respectively (57). The C-terminal CCPs 3–5 are virtually identical to the C-terminal domains of FHR-4A and FHR-4B (93–100%). FHR-3 contains four *N*-linked glycosylation sites, and it circulates in plasma as multiple glycosylation variants ranging from 37 to 50 kDa. A quantitative FHR-3-specific immunoassay was first described by Pouw et al., reporting levels of 0.38 and 0.83 µg/mL for healthy individuals carrying either one or two *CFHR3* copies, respectively (58). These results were later confirmed in a similar assay, reporting mean levels of 1.06 µg/mL (47). Two major genetic variants of *CFHR3* (CFHR3*A and CFHR3*B) have been described (59); interestingly, these are quantitative variants, with CFHR3*B determining higher FHR-3 levels than CFHR3*A (60). The FHR-3*A and FHR-3*B allotypes differ at aminoacid 241 in CCP3 (Pro/Ser), but its functional relevance has not been determined.

### Factor H-Related Protein 4

*CFHR4* is the only known *CFHR* gene that expresses two splice variants, FHR-4A and FHR-4B (61, 62). FHR-4A is composed of nine CCP domains (86 kDa), while FHR-4B has five CCP domains (43 kDa). All FHR-4B domains are also present in FHR-4A, with FHR-4B CCP1 being identical to FHR-4A CCP1, and FHR-4B CCPs 2–5 being identical to FHR-4A CCPs 6–9. FHR-4A CCPs 2–4 seems to have arisen from internal gene duplication, and have high sequence similarity (85–93% amino acid identity) with the other CCPs in FHR-4A/B (61). Thus, obtaining specific reagents to distinguish FHR-4A from FHR-4B is challenging on first sight. Quantification by using an immunoassay that in principle measures both FHR-4A and FHR-4B resulted in average levels of 25.4 µg/mL (27). However, FHR-4A-specific antibodies have been described recently and used in an FHR-4A-specific ELISA which shows 10-fold lower levels for FHR-4A (2.55 ± 1.46 µg/mL) (63). In line with the complete sequence identity of FHR-4B with several FHR-4A domains, no specific antibodies for FHR-4B could be obtained. Strikingly, FHR-4B was not detected in plasma using various antibodies that did react with recombinant FHR-4B (63). This indicates that free FHR-4B must be in an extremely low concentration or even absent from plasma.

### Factor H-Related Protein 5

Factor H-related protein 5 is composed of nine CCPs and is the only FHR with domains (CCPs 3–7) homologous to FH CCPs 10–14 (64). FHR-5 CCPs 1–2 are highly similar (85–93% amino acid identity) to CCPs 1–2 of FHR-1 and FHR-2, although not all residues identified in the FHR-1/2 dimerization motif are present in FHR-5 (26). This could explain why the presence of FHR-5 heterodimers *in vivo* is still controversial (26, 52, 53). FHR-5 seems to circulate predominantly as homodimer *in vivo* (53), making quantification a bit more straightforward. FHR-5 serum levels were reported to be 3–6 µg/mL (24), which was later confirmed in 13 healthy individuals, with median levels of 5.5 µg/mL (65). Similar FHR-5 levels (median 2.46 µg/mL) were found in a larger group of 158 healthy controls using the same immunoassay (55). More recently, an average concentration of 1.66 µg/mL was shown in 115 controls by using a newly developed FHR-5 ELISA (53).

### Other Quantifications

In addition to the specific immunoassays described above, mass spectrometry has also been used to quantify the FHR proteins (66). While this approach allows specific measurement of FHRs based on unique peptide sequences, quantification of FHR dimers is not possible. Results similar to the immunoassays were obtained for FHR-2 (3.64 ± 1.2 µg/mL), FHR-4A (2.42 ± 0.18 µg/mL), and FHR-5 (5.49 ± 1.55 µg/mL). However, much lower levels were found for FHR-1 (1.63 ± 0.04 µg/mL) and FHR-3 (0.020 ± 0.001 µg/mL). It is unclear why such lower concentrations were found for FHR-1 and FHR-3, although the frequency of Δ*CFHR3–CFHR1* in the studied population (*n* = 344, Icelandic origin) was not determined. Of note, the peptide used for FHR-4 quantification is only present in FHR-4A, thus providing no extra information whether FHR-4B exists *in vivo*.

Kopczynska et al. measured FHR-1, FHR-2, and FHR-5 altogether in one immunoassay, finding a total FHR-1/2/5 concentration of 10.67 µg/mL (±5.42) in 42 healthy individuals (67). This result is in great contrast to previously reported levels of approximately 100 µg/mL for FHR-1 (54, 55), but is comparable to a combined mean FHR-1/2/5 concentration of 19.27 (53) and 10.76 µg/mL (66).

The reasons for the huge differences in FHR levels outlined above are unclear. Moreover, the existence of homo- and heterodimers, and the fact that the frequency of the Δ*CFHR3*–*CFHR1* polymorphism is highly population-dependent (19, 68, 69), further complicate the accuracy and assessment of measurements. To exclude any possible cross-reactivity that interferes with FH or FHR quantifications, it is crucial to extensively characterize antibodies generated against FH or any of the FHRs. FH immunoassays should ideally use at least one antibody targeting an epitope located in domains absent from the FHRs, such as CCPs 15–17. Furthermore, when quantifying FHR proteins, it is highly recommended to stratify protein levels based on *CFHR* CNPs, as well as distinguishing between hetero- and homodimers. This would aid in comparison of control and patient groups, as CNP frequencies and dimer formation might be altered in patients. CNPs should be determined at the genetic level, as stratification based only on protein levels seems not to be possible due to the wide range in protein concentration within each CNP group (53, 58). CNPs are most commonly determined using multiplex ligation-dependent probe amplification (MLPA), although there is currently no commercial kit available that also covers *CFHR4*. In addition, while normal levels of FHR proteins are now being reported, further data are necessary to reach consensus on their actual concentrations in circulation.

## Ligands of FH and the FHR Proteins and their Relevance

As outlined above, FH is a major inhibitor of the alternative pathway in plasma and when bound to cells and surfaces like the glomerular basement membrane. This complement regulatory activity is due to the interaction of FH with C3b (70). In addition, FH binds to several other ligands (Figure 2) and, when ligand-bound, in many cases maintains its complement inhibitory activity. These FH interactions ensure proper regulation of complement activation, as well as the resulting opsonization and inflammation.

**Figure 3:**
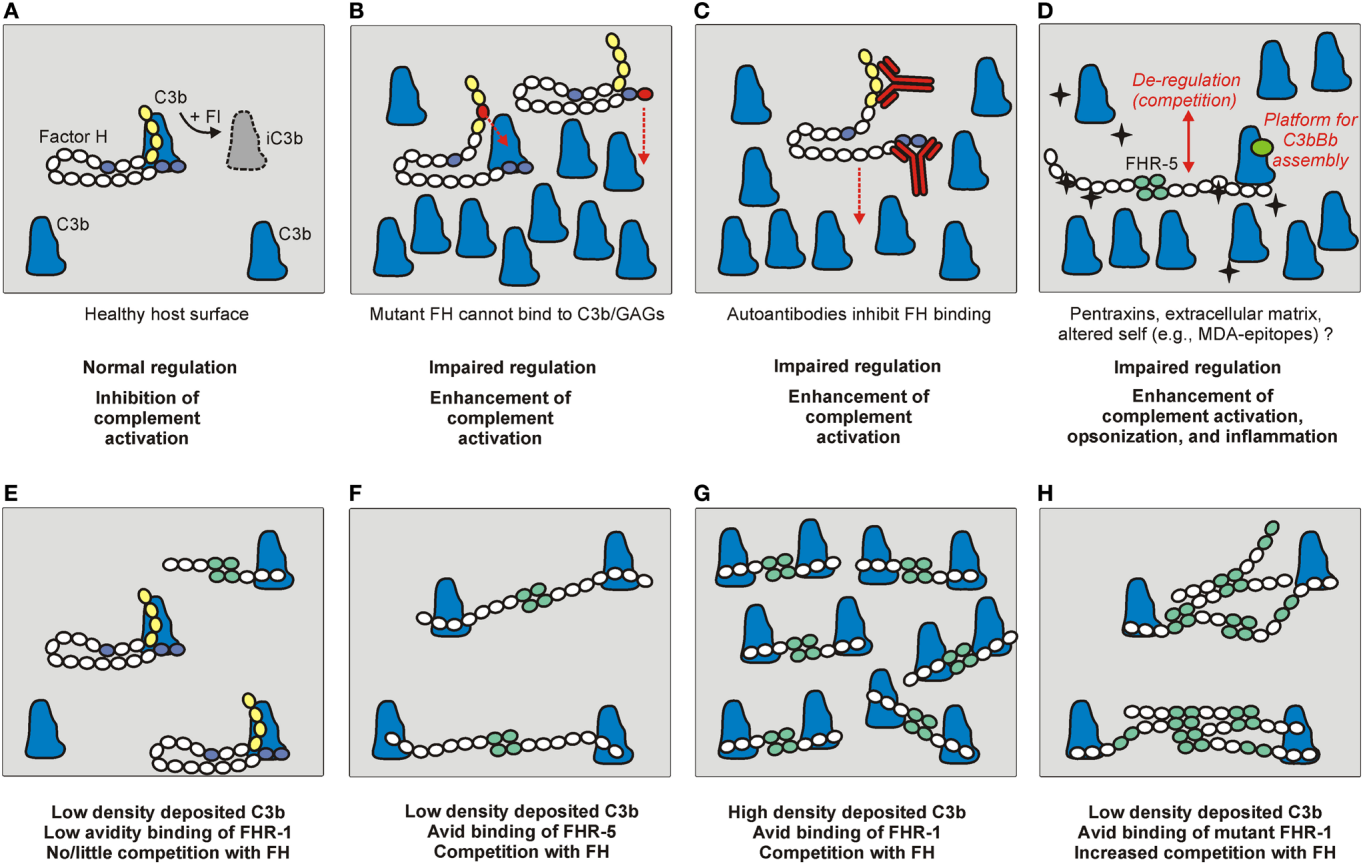
The role of factor H (FH) and the FHRs under physiological and pathological conditions. **(A)** Normal complement control on a healthy host surface by FH bound to surface-deposited C3b. FH recognizes self surfaces under complement attack by binding to a complex of GAGs/sialic acid and C3b. This recognition is mediated by complement control proteins (CCPs) domain 19–20 indicated in blue. Through the activity of the N-terminal regulatory domains (CCPs 1–4, in yellow), FH assists factor I (FI) in the proteolytic cleavage of C3b into inactive C3b (iC3b). **(B)** Mutations in the regulatory or recognition domains, and **(C)** autoantibodies bound to these domains can cause functional FH defect and result in impaired surface complement control. **(D)** FHRs can also interact with similar surfaces and ligands as FH, and compete with FH for binding (de-regulation), and/or they can directly activate the alternative pathway by binding C3b and serving as a platform for the assembly of an active C3bBb convertase. The black star-shape indicates newly exposed ligand/altered self. **(E–H)** The relative FH/FHR concentrations, the ligand density (avidity), and the dimeric/oligomeric states of the FHRs influence surface complement regulation. **(E)** At low ligand (e.g., deposited C3b) density and relative FH surplus, FH can potently regulate complement activation on the surface. **(F)** Increased FHR levels and/or **(G)** ligand densities, and **(H)** formation of higher order oligomers (e.g., due to duplicated dimerization domains) can cause enhanced competition with FH and tip the balance to increased complement activation.

Complement activation can be initiated on modified, dangerous self surfaces, which are recognized by PRMs within (C1q, ficolins, MBL, and properdin) and outside the complement system (e.g., pentraxins). FH along with other regulators may ensure targeted but restricted complement activation and an optimal degree of opsonization, while preventing overt inflammation and damage resulting from cascade over-activation (71, 72). The FHR proteins appear to counter-balance this activity of FH and enhance complement activation by binding to the same or similar ligands and outcompeting FH (Figure 3), and in some instances also by interacting with C3b and other ligands independent of FH (31). This section briefly summarizes the main ligand interactions of FH and the FHR proteins (Figure 2), and indicates their relevance in the regulation and modulation of complement activation.

We would like to briefly note that tumor cells and microbes can bind FH in an attempt to avoid their destruction by host complement. In addition, the main microbial ligand binding sites of FH are in CCPs 6–7 and 19–20, and homologous domains are conserved in the FHRs, thus these proteins may modulate opsonization/killing of microbes. These aspects have been reviewed in detail (6, 31, 73–75).

### C3b

The main ligand of FH is the active C3 fragment C3b, which can be generated by fluid phase and surface-bound C3 convertases. Since C3b is the central component that promotes complement amplification *via* the alternative pathway, and is also required for the assembly of C5 convertases and the initiation of the terminal pathway, its regulation is key to maintain the proper balance of complement activation and inhibition. FH interacts with C3b at two main sites, harbored by CCPs 1–4 and 19–20 (76). The N-terminal C3b binding site is active when FH is in the fluid phase (e.g., in blood plasma) and also when FH is bound to cells or other surfaces [*via* glycosaminoglycans (GAGs), sialic acid, or a specific receptor—see below] (Figure 3A). FH may also bind C3b by CCPs 1–4 when already bound to other ligands, such as pentraxins, because these interactions typically involve CCPs 6–7 and 19–20 (74, 77–79). Thus, FH maintains its complement regulatory activity when bound to cells or other ligands.

Structural studies revealed that FH engages surface-deposited C3b in the context of host GAGs/sialic acid, i.e., CCPs 19–20 bind to these ligands at the same time, which allows avid interaction of FH with a host surface under complement attack. The FH C-terminal site also binds C3d, the final C3b degradation product that remains covalently attached to the surface (80, 81).

The FHR proteins also bind to C3b, but the nature of these interactions is inherently different from that of FH because FHRs lack domains homologous to FH CCPs 1–4. Thus, FHRs lack FH-like cofactor activity and decay accelerating activity, although some residual activity may be present due to the interaction of the C-terminal domains of these proteins with C3b. This should be investigated in detail in the future to clarify the currently contradicting reports in this regard (20, 23, 24, 26, 27, 32, 33).

In contrast to possible inhibitory activities, FHR-1, FHR-4, and FHR-5 were reported to activate the alternative pathway, by binding C3b through their C-terminal domains and forming a platform for the assembly of an active C3bBb convertase (27, 32, 33). This activity could take place on surfaces where these FHRs are bound directly, or *via* another ligand, such as pentraxins (33). FHR-1 and FHR-5 were shown to enhance complement activation on the extracellular matrix (ECM) and on the surface of apoptotic or necrotic cells (32, 33, 82).

Additionally, FHRs may compete with FH for binding to C3b deposited on surfaces, a process termed complement de-regulation, because FHRs can enhance complement activation by inhibiting FH binding (Figure 3D). This activity of the FHRs may only be significant—considering their relative serum concentrations and avidity for C3b—if increased amounts of FHRs or altered FHR forms (such as higher order oligomers) are present (Figures 3E–H) (25, 26, 52, 83, 84). For FHR-2, it was described that, despite binding to C3b, it cannot effectively compete with FH for binding to surface-bound C3b (20).

Altogether, based on these data the FHRs can be regarded as positive complement regulators.

### Other C3 Fragments

While interacting sites for other C3 fragments were described, current evidence strongly supports the physiologically relevant binding of FH to C3b *via* CCPs 1–4 and 19–20, as well as to C3d *via* CCPs 19–20 (76). Interaction of FHR-1 and FHR-2 with C3d was also shown, but without functional analyses (20, 84). Binding of FHR-3, but not of FHR-1, to C3d was shown to prevent the binding of C3d to its receptor on B cells, thus modulating B cell activation (85). FHR-5 was reported to bind to iC3b and C3d with affinities similar to C3b; in contrast, FH bound very weakly to iC3b and C3d compared with FHR-5, indicating that despite its lower serum concentration FHR-5 can be an efficient competitor of FH for binding to deposited C3 fragments (26).

### Glycosaminoglycans (GAGs), Sialic Acid, and Heparin

Distinction between self, non-self, or altered self surfaces relies in part on the recognition of host-specific GAGs and sialic acid by FH (and FHL-1). This allows complement activation to proceed unhindered on microbial (“activator”) surfaces, but prevents activation on host (“non-activator”) surfaces (86). This has been a subject of intensive research, often using heparin as a model for polyanionic molecules. The main heparin-binding sites were identified in FH (and FHL-1) CCP7 and FH CCP20 (87, 88). This allows recognition of, and attachment to, host glycomatrix and cells, such as platelets and endothelial cells (89, 90). Recent studies revealed some functional differences indicating that while some GAGs are recognized by FH and FHL-1 *via* CCP7, the sialic acid binding site is in CCP20 (91), also targeting these host regulators to different surfaces and explaining the different consequences of mutations affecting these domains (89, 92, 93).

Factor H-related protein 1 can also bind to host surfaces *via* its FH-homologous C-terminus (22, 29), and FHR-3 binds heparin through CCP2, which is homologous to CCP7 of FH (23, 87). In addition, FHR-5 has a heparin-binding site in CCPs 5–7 (24, 94). The functional relevance of these interactions needs to be investigated further, but they could anchor these proteins on certain cells and surfaces.

### ECM as a Non-Cellular Surface

Extracellular matrices occur in many tissues and can have different functions, the most important ones being the physical support of cells and acting as barriers and filters. The composition of ECMs differs at distinct anatomic sites and is dynamically regulated. Under certain conditions, e.g., endothelial cell activation or injury, ECMs can be exposed to body fluids and plasma proteins; in addition, the Bruch’s membrane in the eye and the kidney glomerular basement membrane are also exposed because the lining cell layer is fenestrated. To prevent overt complement activation, such ECMs rely largely on soluble complement regulators, such as FH and FHL-1, which can bind *via* their GAG binding sites and locally regulate complement (95). As noted above, differences in ECMs and in domain composition of the FH family proteins may target FH and FHRs toward distinct sites, such as FH to the glomerular basement membrane (*via* CCPs 19–20) and FHL-1 to the Bruch’s membrane (*via* CCP7) (95). FHR-5 was shown to bind to MaxGel, an ECM extract, and de-regulate complement on this surface (32); a recent study identified laminin as an ECM ligand of FHR-5 (94).

### Ligands on Dead Cells

Complement is largely involved in the immunologically safe and silent disposal of apoptotic and necrotic cells *via* opsonophagocytosis (96). The soluble regulators FH and C4b-binding protein bind to dead cells and prevent excessive complement activation and potential deleterious effects when membrane-anchored regulators are down-regulated on the cells (97). FH can bind to Annexin-II, DNA, and histones (98), as well as malondialdehyde epitopes on apoptotic cells (94, 99). In addition, the pentraxins CRP and pentraxin 3 (PTX3) also bind to dead cells and recruit FH (77, 100). For FHR-1 and FHR-5, binding to necrotic cells and enhancement of complement activation have been shown (33, 82), suggesting that these FHRs modulate opsonization of dead cells.

### Pentraxins

The pentraxins are soluble PRMs of the innate immune system and, based on their structure, categorized as short and long pentraxins. Pentraxins have numerous ligands and functions, reviewed in detail elsewhere (101); of note, they participate in the opsonization of microbes and dead cells, and they also bind to components of the ECM. For the prototypic short pentraxin CRP and the long pentraxin PTX3, interactions with both complement activators (C1q, MBL) and inhibitors (FH, C4b-binding protein) were described (74, 77, 79, 101–107).

C-reactive protein circulates in its native, pentameric form (pCRP) in body fluids, but it can adopt an altered conformation exposing neoepitopes upon pH change or binding to membranes, and it can even decay to its monomeric form (mCRP) *in vitro* by chelation of the Ca^2+^ ions or adsorption on plastic. FH was described to bind primarily to mCRP *via* CCPs 7, 8–11, and 19–20 (79, 108, 109), but interaction with pCRP *via* CCPs 7 and 19–20 at acute phase concentrations was also reported (110). The binding to mCRP allows targeting of the complement inhibitor FH to certain surfaces, including apoptotic cells (71, 100, 109). Among the FHRs, FHR-1 binds to mCRP *via* CCPs 4–5 (33) and FHR-5 *via* CCPs 5–7 (24, 32). The FHR-1/mCRP interaction enhanced classical and alternative pathway activation, and FHR-5 efficiently competed with FH for mCRP binding, resulting in enhanced complement activation on mCRP (32, 33). In contrast, FHR-4 binds to pCRP *via* CCP1, and this interaction results in enhanced classical pathway activation (111, 112).

PTX3 forms a complex, octameric structure stabilized in part by covalent bonds (113). PTX3 binds to FH *via* CCPs 7 and 19–20, and recruits it to apoptotic cells to downregulate complement activation (77). PTX3 also binds to FHR-1 (weaker than FH) and FHR-5 (stronger than FH); FHR-5 competes with FH and enhance complement activation on PTX3 (32, 33, 74).

### Malondialdehyde Epitopes

Malondialdehyde (MDA) and malondialdehyde-acetaldehyde (MAA) adducts of proteins and lipids may be generated upon oxidation as oxidation-related neoepitopes, and induce inflammatory responses. FH was shown to bind to MDA/MAA epitopes and inhibit complement activation and the proinflammatory effects of such MDA/MAA epitopes (99). Two binding sites, within CCP7 and CCPs 19–20 of FH, were identified to bind to MDA/MAA epitopes (99, 114). Recently, FHR-5 was also shown to bind to MAA epitopes (MAA-BSA) *via* CCPs 5–7 and to compete with FH for MAA-BSA binding, thus increasing complement activation. In addition, binding of FHR-5 to necrotic cells was mediated by the same domains, possibly in part *via* the MDA/MAA epitopes that appear on dead cells (94).

### Other, Less Characterized Ligand Interactions of FH

Factor H binds to other ligands that are implicated in certain diseases, particularly in the thrombotic microangiopathy atypical hemolytic uremic syndrome (aHUS). One of these ligands is thrombomodulin, a transmembrane glycoprotein present in endothelial cells, which is involved in the regulation of coagulation and inflammation; thrombomodulin soluble fragments can also be released upon endothelial cell activation or injury. Thrombomodulin was shown to bind to FH and the FH–C3b complex with nanomolar affinity and to enhance FH cofactor activity, which would be reduced in the case of thrombomodulin mutations in aHUS (115–117). These data suggest a role for thrombomodulin in inhibiting alternative pathway activation locally *via* its interaction with FH, but thrombomodulin was also found to inhibit complement hemolytic activity in a FH-independent mechanism (116). An additional, complement-activating function of thrombomodulin by enhancing C3 cleavage into C3b has also been described (117).

Similarly, binding of von Willebrand factor (vWF) to FH enhances FH cofactor activity and also modulates the vWF prothrombotic status (118–120). FH was found co-localized with vWF in the Weibel–Palade bodies in human umbilical vein endothelial cells, and the complex was also detected in human plasma. Purified FH and vWF were shown to interact with nanomolar affinity, and to influence their respective functions; vWF enhanced the cofactor activity of FH, whereas FH inhibited ADAMTS13-mediated cleavage of vWF and facilitated platelet aggregation (120). However, another investigation found that FH binds *via* its C-terminus to the vWF A2 domain, and enhances its cleavage by ADAMTS13 (118). FH was also reported to reduce large soluble vWF multimers (119). Thus, further studies are needed to clarify the functional relevance of the complex interaction between FH and vWF, and its potential role in disease.

Recently, hemolysis-derived heme was shown to activate the alternative pathway in serum and on endothelial cells, and to bind both C3 and FH. Heme-exposed C3 and endothelial cells displayed increased FH binding, and FH was shown to be a major serum factor that regulates C3 deposition on heme-treated endothelial cells (121).

Factor H was also reported to bind to apolipoprotein E *via* domains CCPs 5–7, and to regulate alternative pathway activation on high density lipoprotein particles (122). Complement regulation by FH on such lipoprotein particles could be potentially impaired in diseases characterized by immune deposits containing also apolipoprotein E, such as age-related macular degeneration (AMD) and dense deposit disease (DDD) (122).

In addition, FH binds to myeloperoxidase (MPO) released from activated neutrophil granulocytes, and FH and MPO co-localize in neutrophil extracellular traps. Interestingly, the binding site for MPO in FH was determined to be CCPs 1–4 and, thus, MPO inhibited FH binding to C3b, as well as FH decay accelerating activity and cofactor activity (123).

### Binding to Cellular Receptors—Non-Canonical Roles of the FH Family Proteins

Factor H and some of the FHRs can also bind to cells *via* specific receptors, and may modulate the cell activation and response, as well as inflammatory processes. These aspects are reviewed in detail elsewhere (124); here, we summarize only some major FH/FHR-receptor interactions and their role, particularly those described very recently.

Complement receptor type 3 (CR3; CD11b/CD18; or integrin α_M_β_2_) was identified as a main FH receptor on neutrophils and macrophages (125, 126). FH maintains its cofactor activity when receptor bound, but it also directly affects cellular functions, such as adhesion, cell spreading, migration, and cytokine production (125–127). Interestingly, FH was able to inhibit the release of extracellular traps by human neutrophils activated with immobilized fibronectin plus fungal β-glucan, or with phorbol 12-myristate 13-acetate (127). FH can also enhance the interaction of certain pathogens with human macrophages and neutrophils, and modulate the response of the phagocytes (128, 129). This was also shown for FHR-1 which, by binding to CR3, could enhance neutrophil responses to *Candida albicans* (129). In addition, FHL-1 was shown to mediate cell adhesion and spreading (129, 130).

Described functional effects of FH on monocytes include enhancement of IL-1β secretion, respiratory burst, and chemotactic effect (131–134). FH was shown to induce an anti-inflammatory and tolerogenic phenotype in monocyte-derived dendritic cells *in vitro* (135). Very recently, in the context of inflammation in AMD, FH, and its two variants Y402 and H402 were investigated in a mouse model. FH was shown to inhibit the resolution of inflammation by binding to CR3 and thus blocking thrombospondin-1–CD47 signaling that would normally promote the elimination of macrophages. The AMD-associated H402 FH variant displayed a stronger inhibitory effect compared to FH Y402, causing increased accumulation of macrophages in the inflamed tissue (136).

Factor H was shown to bind to B cells and may modulate some B cell functions, such as proliferation and immunoglobulin secretion, but no specific receptor has been identified to date (137–140). A recent report described an indirect modulation of B cell activation by FHR-3, which was shown to bind to C3d and inhibit its binding to complement receptor type 2, a co-receptor of the B cell receptor complex; FH and FHR-1 had no such effect (85).

These non-canonical functions of the FH family proteins deserve further investigation, because they may play roles in inflammation and anti-microbial defense that are currently underappreciated. Clarification of their cell-mediated effects may provide additional insights into disease mechanisms.

## Disease Associations

Studies in patients and controls have shown a variety of common *CFH/CFHRs* genetic variants that predispose to autologous damage, which is predominantly organ-specific. Prevalent kidney damage occurs in the rare diseases aHUS and C3 glomerulopathies (C3G), and in the more frequent IgA nephropathy (IgAN), while destruction of the retinal pigment epithelium by autologous complement contributes to AMD. A defective regulation of complement activation on the renal microvasculature endothelium occurs in aHUS, while in C3G uncontrolled complement activation in plasma gives rise to massive deposition of C3b breakdown products (iC3b, C3dg, and C3c) in the glomeruli (141–143). IgAN is characterized by mesangial cell proliferation and hypoglycosylated IgA1 deposits in the glomeruli, and it is likely that complement defects contribute, at least in part, to its clinical heterogeneity (144, 145). A defective control of complement activation in the retina is most relevant in AMD pathogenesis, and enhances the inflammatory response (146).

Extremely rare and pathogenic *CFH/CFHRs* variants have been mainly found in aHUS and C3G patients. Some of these variants result from gene conversion events between *CFH* and *CFHR1*, and they give rise to mutated FH or FHR-1. Other variants are intragenic duplications or hybrid genes resulting from gene rearrangements, and generate abnormal proteins; some of these proteins have distinct molecular weights and can be detected by Western blot analysis. It is interesting that abnormal rearrangements involving FH/FHRs associate with aHUS, while in C3G patients only FHR proteins are affected.

### *CFH* Variants Associated With Renal or Ocular Damage

Common SNPs in *CFH* give rise to different haplotypes that can be disease neutral, predisposing, or protecting. Thus, haplotype *CFH(H1)* predisposes to membranoproliferative glomerulonephritis (MPGN) and AMD, haplotype *CFH(H3)* predisposes to aHUS, and haplotype *CFH(H2)* is protective against these three diseases (147–149). Haplotype *CFH(H2)* generates the FH_62Ile_ variant, which shows increased binding to C3b and cofactor activity in the fluid phase and on cellular surfaces (150), thus favoring protection against autologous complement damage.

The common variant FH_402His_, which is present in FH and its shorter isoform FHL-1, is a major predisposing factor in AMD (151). The functional relevance of FH_402His_ in C3b, CRP, or heparin binding has been analyzed in several studies. Reduced binding of FH_402His_ to polyanionic surfaces has been found (152), but the pathogenic mechanism may also depend on FHL-1, which can regulate complement activation similarly to FH. It has been shown that FHL-1, but not FH, is present in the retinal Bruch’s membrane, a major target in AMD pathogenesis, and that binding of the AMD-FHL-1_402His_ variant was lower than binding of the FHL-1_402Tyr_ variant (93). Nonetheless, *CFH* intronic variants show stronger association with AMD than FH_402His_ (153). In an analysis of seven common *CFH* haplotypes, haplotypes H1, H6, and H7 were found to confer increased risk to AMD; these haplotypes share a 32-kb region downstream of rs1061170 (FH Tyr402His) that must be critical for AMD development (19), and that includes a 12-kb block 89% similar to a noncoding region in CNP148 (see below).

Other disease-predisposing FH variants are very rare. One of the most relevant is Arg1210Cys (FH_1210C_), which was initially identified in aHUS patients (154), and shown to be covalently bound to albumin in plasma (155); the presence of albumin most likely prevents the interaction of FH_1210C_ with its physiological ligands, generating a partial, pathogenic FH deficiency. FH_1210C_ has been also associated with C3G (156), and it highly increases AMD-risk and predisposes to early disease onset (157, 158). It has been suggested that in individuals with the FH_1210C_ variant it is the concurrence of other genetic predisposing factors what ultimately determines the clinical phenotype (159).

### *CFHR1* and *CFHR3* Variants Associated With Renal or Ocular Damage

As happens with the common *CFH* haplotypes, the two main *CFHR1* alleles show differential disease associations. CFHR1*B, displaying increased similarity with *CFH*, increases aHUS risk (51), and CFHR1*A predisposes to AMD (160). The molecular bases for these associations have not been determined, but they will most likely depend on subtle functional differences among the FHR-1*A and FHR-1*B allotypes. CFHR1*A is in strong linkage disequilibrium with the AMD-risk *CFH402His* allele, and *CFHR1* genotyping has similar predictive value of developing AMD as *CFH402His;*Δ*CFHR3–CFHR1* genotyping (160); these findings are suggestive of a direct role of FHR-1 in AMD pathogenesis, most likely by interfering with the interaction of FH with specific ligands and promoting complement activation (33).

The *CFHR3* gene also has two major variants, CFHR3*A, more frequent in healthy controls, and CFHR3*B, which predisposes to aHUS but not to C3G (59). Because the aHUS risk CFHR3*B allele generates higher FHR-3 levels than the non-risk CFHR3*A allele (60), it seems that increased competition of FHR-3 and FH for certain ligands could favor aHUS development. FHR-3 is also produced in the retina, and its contribution to retinal degeneration by inhibiting FH binding to C3b and modified surfaces has been suggested (47); nonetheless, the relevance of the CFHR3*A and CFHR3*B variants in AMD has not been addressed.

The two CNPs in the *CFHR* genes have been shown to be disease-relevant (19). The common variant Δ*CFHR3–CFHR1* is protective against AMD (161), and IgAN (162), but it predisposes to aHUS (163) and to systemic lupus erythematosus (SLE) (69) because it is associated with generation of anti-FH autoantibodies (discussed on page 13). The rare variant Δ*CFHR1–CFHR4* was initially identified in a few aHUS patients with anti-FH autoantibodies (51), and is present in 1.4% of aHUS patients and 0.9% of controls (164).

The protective effect of the Δ*CFHR3*–*CFHR1* haplotype against AMD was first described in 2006 (161), and it is the more common copy number variation in the *CFH/CFHRs* region (165). Δ*CFHR3*–*CFHR1* is tagged by *CFH* rs6677604A with 99% accuracy (166), and strongly correlates with the 86.4-kb deletion CNP147 and high FH levels (8). Because protection conferred by Δ*CFHR3*–*CFHR1* was independent of the FH Tyr402His polymorphism, a direct effect of FHR-1 and FHR-3 in AMD pathogenesis was suggested (21). Nonetheless, the strong association of Δ*CFHR3*–*CFHR1* with high FH levels, together with the finding that FHR-1 levels were lower in AMD patients than in control individuals, suggests that Δ*CFHR3*–*CFHR1* is actually tagging an allele expressing high FH levels, but it is not causal in protection against AMD (8). The much rarer Δ*CFHR1*–*CFHR4* deletion (also referred to as CNP148) also confers protection against AMD independent of SNPs in *CFH* (19); because Δ*CFHR1*–*CFHR4* also removes non-coding flanking regions, its protective effect against AMD could either be due to the reduction of FHR-1 and/or FHR-4A levels, or to the absence of regulatory regions relevant for disease pathogenesis.

The first evidence for a direct complement role in IgAN pathogenesis was the finding that the common variant Δ*CFHR3*–*CFHR1* protects against IgAN when it is in homozygosis (162), pointing out to a possible role of FHR-3 and/or FHR-1 levels in the pathogenic mechanism. However, because the Δ*CFHR3*–*CFHR1* allele generates high FH levels which associate with lower mesangial C3 deposition, the actual contribution of FHR-1 and/or FHR-3 levels to IgAN is unclear (167). Two studies in different IgAN cohorts have recently shown that FHR-1 levels and FHR-1/FH ratios are increased in patients with disease progression, thus providing evidence for a direct role of FHR-1 in the disease mechanism. One of these studies reported that high FHR-5 levels were also slightly elevated in the IgAN patients, but without any correlation with progressive disease (55). The other study also reported low FH levels associated with *CFH* or *CFI* mutations in a few IgAN patients (54).

### FH::FHR-1, FHR-1::FH, and FH::FHR-3 Hybrid Proteins Associate With aHUS

*CFH* exons 18–20 and *CFHR1* exons 4–6 have a high degree of sequence similarity, that result in only five amino acid difference between CCPs 18–20 of FH (_Y1040-V1042-Q1058-S1191-V1197_) and CCPs 3–5 of FHR-1 (_H157-L159-E175-L290-A296_). Studies in aHUS patients have revealed that these differences determine higher binding of FH than FHR-1 to cell surfaces. Amino acids S1191 and V1197 in FH seem to be particularly important, and single mutations involving these amino acids (FH_S1191L_ and FH_V1197A_) have been found in a number of aHUS patients from different geographical origins. The double mutant (FH_S1191L-V1197A_) was observed in two unrelated aHUS patients with early disease onset, showed a defective capacity to control complement activation on cellular surfaces, and had been generated by gene conversion (168).

FH_S1191L-V1197A_ can also be generated by NAHR events that give rise to *CFH::CFHR1* hybrid genes. A *CFH(Ex1*–*21)::CFHR1(Ex5*–*6)* hybrid gene was first described in a family with many cases of aHUS along several generations, and a clinical history of disease recurrence in affected individuals (169), demonstrating that FH_S1191L-V1197A_ is highly pathogenic. This hybrid gene has also been found in other non-related aHUS patients. A slightly different *CFH(Ex1*–*22)::CFHR1(Ex6)* gene which also generates FH_S1191L-V1197A_ has been found in another patient with a prompt aHUS onset (170). The reverse situation (i.e., the existence of *CFHR1::CFH* hybrid genes) has also been reported. A *CFHR1(Ex1*–*3)::CFH(Ex19*–*20)* hybrid gene generated by “*de novo*” NAHR was identified in one sporadic case of aHUS (171), and a *CFHR1(Ex1*–*4)::CFH(Ex20)* hybrid gene was found in a family with two members affected with aHUS (172). These two *CFHR1::CFH* hybrid genes generated a double-mutated FHR-1 protein that carries the homologous amino acids in FH CCP20 domain (FHR-1_L290S-A296V_); these amino acids most likely confer the mutated FHR-1 increased competition with FH for endothelial cell binding, and result in reduced protection against complement damage (173). Screening of *CFH::CFHR1* and *CFHR1::CFH* hybrid genes is normally done by MLPA analysis of copy number variations. The *CFH::CFHR1* alleles lack a normal copy of *CFHR3* and *CFHR1*, while the *CFHR1::CFH* allele contains two copies of *CFHR3*; it cannot be ruled out that these additional factors also contribute to the pathogenic mechanism.

A FH::FHR-3 hybrid protein containing CCPs 1–19 of FH and the five CCPs of FHR-3 was identified in a large family with aHUS (174). This protein resulted from an abnormal rearrangement that deleted the last exon of *CFH*, which was then fused to the adjacent *CFHR3* gene by the genetic mechanism microhomology mediated end joining (MMEJ). The absence of FH CCP20 domain in the hybrid protein and/or the presence of the FHR-3 CCPs does not affect complement regulation in the fluid phase, but cellular surface regulation seemed to be highly reduced. Estimation of aHUS penetrance in carriers of the hybrid gene is 33%. Another FH::FHR-3 hybrid protein containing CCPs 1–17 of FH and the five CCPs of FHR-3 was found in an aHUS patient with a very early disease onset (175). The hybrid protein resulted from a *“de novo”* 6.3 kb-deletion of exons 21–23 of the *CFH* gene through a MMEJ mechanism, and it showed impaired cell surface complement regulation.

### Abnormal FHR Proteins in C3G

The abnormal rearrangements that predispose to C3G thus far described involve exclusively the *CFHR* genes, but not the *CFH* gene. This is a distinctive feature from aHUS that suggests a more important contribution of FHRs in the protection of the glomerular basement membrane and mesangium than in protection of endothelial cells. Abnormal rearrangements include intragenic duplications in *CFHR1* or *CFHR5*, and *CFHR2::CFHR5* and *CFHR3::CFHR1* hybrid genes.

### FHR-5 and FHR-1 Proteins With Additional Dimerization Domains

Larger forms of FHR-1 and FHR-5 with duplicated dimerization domains have been observed in a few C3G patients. These proteins circulate in plasma together with the normal FHR-1 and FHR-5 proteins, but disease penetrance in mutation carriers is very high, strongly suggesting a dominant negative effect of the larger, abnormal protein. This is particularly evident for a partially duplicated FHR-5 protein initially observed in two families of Cypriot ancestry in which renal disease was consistent with autosomal dominant transmission (176). All affected individuals were heterozygous for a *CFHR5* gene in which exon 2 (coding for CCP1) and exon 3 (coding for CCP2) were duplicated, giving rise to an abnormal FHR-5 protein containing two extra dimerization domains (FHR-5_12123-9_). *In vitro* studies with patient’s sera showed reduced binding of the FHR-5_12123-9_ to the cell surface, and increased FI cofactor activity, but the relevance of these findings for the pathogenic mechanism is unknown. Patients carrying FHR-5_12123-9_ had a high risk of progressive renal disease, particularly males. This renal phenotype, which histologically corresponds to a C3 glomerulonephritis, is clinically characterized by continuous macroscopic hematuria, and was denominated as “CFHR5 nephropathy.” These seminal observations were further extended to 16 pedigrees of Cypriot origin in a study that also provided a thorough description of histological, molecular, and clinical findings (177). Recurrence of CFHR5 nephropathy in a kidney allograft has been reported in one patient, although it did not occur in two other cases (178). The same duplicated FHR-5 protein observed in patients of Cypriot ancestry was found in a familial case of C3 glomerulonephritis with a different ethnic origin (179). Of note, this protein was generated from a different genomic rearrangement, reinforcing the relevance of the duplicated FHR-5 protein for the pathogenic mechanism, and the authors proposed that all patients with clinical suspicion of CFHR5 nephropathy should be screened for the abnormal protein by Western blot.

Another FHR-5 protein with two additional dimerization domains was found in a familial case of C3G with DDD (C3G-DDD) (83). In this family, a genomic 24.8 kb-deletion from intron III of the *CFHR2* gene to *CFHR5* gives rise to a hybrid *CFHR2::CFHR5* gene which generates a so-called FHR-2_1,2_-FHR-5-hybrid protein very similar to the FHR-5_12123-9_ protein previously described. This hybrid protein shows increased binding to C3b and stabilization of the AP C3 convertase, which would explain the low C3 and increased Ba levels detected in the patients’ sera; in addition, reduced regulation of the AP C3 convertase by FH will result in increased generation of iC3b molecules which will deposit on the glomerular basement membrane and favor the pathogenic mechanism.

To understand how the presence of two extra dimerization domains in FHR-5_12123-9_ has pathogenic consequences it is necessary to recapitulate that the dimerization domains in FHR-1, FHR-2, and FHR-5 confer these proteins the ability to generate homo- and hetero-dimers physiologically (26). Although a recent study has not found evidence of the presence of FHR-5 heterodimers with FHR-1 or FHR-2 (53), the additional dimerization domains in FHR-5_12123-9_ and FHR-2_1,2_-FHR-5 will most likely give rise to higher order oligomeric forms with increased avidity for surface-bound C3b, and these multimeric proteins will compete more efficiently with FH and favor autologous tissue damage, as illustrated in Figure 3. In this context, it is intriguing that two very similar FHR-5 proteins result in different clinical entities (CFHR5 nephropathy or DDD). FHR-5_12123-9_ and FHR-2_1,2_-FHR-5 contain the nine CCPs of FHR-5 preceded by the two dimerization domains of FHR-5 or FHR-2, respectively, that present 85% aminoacid identity. Functional studies with the recombinant forms of these two proteins (referred to as FHR-5_Dup_ and FHR-2-FHR-5_Hyb_) revealed that they exacerbate local complement activation by recruiting the complement-activating protein properdin, and that properdin binding is mediated by the FHR-5 dimerization domains, and not by the FHR-2 dimerization domains (82). Therefore, local complement activation would be higher in patients with FHR-5_Dup_ than in patients with FHR-2-FHR-5_Hyb_, and this could explain the different clinical phenotype. Another, non-exclusive, explanation is that the pathogenic mechanism is much dependent on the plasma levels of these FHR-5 proteins. In line with this hypothesis, Western blot analyses of patient serum samples showed that the FHR-5 band has similar intensity to a normal serum, while the intensity of the FHR-5_12123-9_ (32) or the FHR-2_1,2_-FHR-5 band is much higher, suggesting highly increased levels (82). The latter study also showed that FHR-5 binds to necrotic human endothelial cells, but not to normal endothelial cells, strongly suggesting a role for FHR-5 in complement-mediated elimination of damaged cells.

An abnormal, large FHR-1 protein was identified in a Spanish family with C3G by Western blot analysis (52). This protein was generated by an internal duplication of the *CFHR1* gene, and contains two copies of domains CCPs 1–4. Purification of the normal and the duplicated FHR-1 proteins allowed biochemical, functional, and structural studies that illustrated that normal FHR-1 circulates in plasma as homo- and hetero-oligomers (with FHR-2 and FHR-5), and that the duplicated FHR-1 (containing nine CCP domains) organized into much larger oligomers with increased binding to C3b, iC3b, and C3dg. These findings provided the first evidence for the existence of oligomeric forms of FHR-1, FHR-2, and FHR-5 in normal plasma, and confirmed that duplication of their homologous CCPs 1–2 is pathogenic and associates with C3G. The authors proposed that multimerization of FHR-1 strongly inhibits FH binding to certain cell surfaces, but not to endothelial cells, the target surface in aHUS. A different FHR-1 protein containing two copies of domains CCPs 1–2 has been described in another Spanish patient with a C3G clinical phenotype, but further characterization of this duplicated FHR-1 (containing seven domains) has not been provided (180).

### FHR-3::FHR-1 Hybrid Protein

A hybrid *CFHR3::CFHR1* gene associated with C3G-MPGN III has been described in an Irish family (181). This hybrid gene contains exons 1–3 of *CFHR3* and exons 2–6 of *CFHR1*, and generates a protein containing CCPs 1–2 of FHR-3 followed by the five CCPs of FHR-1. The protein was detected in the patients’ plasma by Western blot, and it was apparently at a much lower concentration than normal FHR-1. Because patients with the *CFHR3::CFHR1* gene also has two copies of *CFHR3* and *CFHR1*, the authors propose a dominant effect of the hybrid FHR-3::FHR-1 protein in the pathogenic mechanism. It is of note that plasma C3 levels in all affected individuals were normal, as opposed to the reduced levels observed in the C3G-DDD patient with FHR-2_1,2_-FHR-5-hybrid protein (83); this fact suggests that the potential pathogenic effect of FHR-3::FHR-1 on complement activation or regulation is surface-restricted. The clinical data and outcome of the five patients from this family who received renal transplantation has been reported (182); disease recurrence in the kidney allograft was high, but the overall graft survival was good.

### Anti-FH Autoantibodies Predispose to Renal Diseases

Disorders related with these autoantibodies are mainly present in aHUS and C3 glomerulonephritis patients and secondary in other autoimmune diseases. The anti-FH autoantibodies cause a functional FH defect, resulting in impaired complement regulation by FH (Figure 3C).

The existence of anti-factor H autoantibodies in aHUS and the resulting functional deficiency of FH were first described in 2005 (183). The frequency of the anti-FH antibodies associated with aHUS is approximately 10% of the pediatric patients in the European series and occasionally in patients with adult onset (184). These autoantibodies form complexes with FH and induce a functional FH deficiency. Characterization of these autoantibodies showed that they recognized the C-terminal region of FH, involved in the binding to cell surfaces (185, 186). Moreover, it has been shown that, especially in the acute phase, these antibodies are also capable of blocking the activity of FH as cofactor of FI and the acceleration of the dissociation of the convertases of the alternative pathway (187).

The presence of anti-FH autoantibodies is associated with homozygous Δ*CFHR3–CFHR1* in several aHUS cohorts (51, 164, 188, 189). The Δ*CFHR1–CFHR4* has also been found in a few patients (51, 164), suggesting a relevant role for the absence of FHR-1 in autoantibody generation. In this context, it has been found that most anti-FH autoantibodies also bind to FHR-1, which presents high similarity with FH CCPs 19–20 (29, 164).

The anti-FH autoantibodies in aHUS patients are able of forming immune complexes that can be detected in serum. The amount of these complexes correlates better with the clinical evolution than the total autoantibody titer (187), because FH bound to the complexes cannot regulate the AP on cell surfaces. The use of two monoclonal antibodies binding to different parts of FH allowed the quantitation of total and free FH, which depends on the concentration of circulating anti-FH immune complexes (29, 186, 190). In some cases, the concentration of total FH was within the normal range, but the amount of free FH was practically undetectable, indicating that the anti-FH autoantibodies almost completely blocked the ability of FH to protect cell surfaces from complement activation, although its regulatory activity in the fluid phase was conserved (190).

The epitope recognized by anti-FH autoantibodies has been defined more precisely using recombinant fragments of CCPs 19–20 containing point mutations (191). In this work, it was found that in patients with FHR-1 deficiency, anti-FH antibodies recognize a region that acquires a different conformation in FH and FHR-1 after binding to certain ligands, including various bacterial proteins. This suggests a model in which the absence of FHR-1 plays a role in the loss of tolerance to FH and in the generation of anti-FH autoantibodies, thus explaining the frequent association between the presence of anti-FH antibodies and homozygous Δ*CFHR3–CFHR1* in aHUS. By using the same mutated FH recombinant fragments in our series of patients with anti-FH autoantibodies, we have obtained concordant results, at least in the patients with FHR-1 deficiency, which supports the proposed model for the generation of autoantibodies in these patients (192). However, the mechanism of anti-FH autoantibody generation in aHUS patients without FHR-1 deficiency remains to be determined.

Anti-FH autoantibodies have also been described in patients with C3G (193–197). This association is much less frequent than in the case of aHUS despite having been described for the first time (196). In cases in which the effect of these anti-FH autoantibodies has been studied, it has been shown that they inhibit the regulatory activity of FH by recognizing and blocking its N-terminal region (193, 194, 197), which is a difference with the anti-FH autoantibodies from aHUS patients.

In patients with SLE and other autoimmune diseases, a greater frequency of anti-FH autoantibodies has been described with respect to healthy controls (198). Unlike the anti-FH autoantibodies present in aHUS, the epitopes that are recognized by the autoantibodies seem to be distributed throughout the entire protein, and they are not associated with FHR-1 deficiency.

## Conclusion

The FH protein family remains an intriguing group of proteins. FH is well-known for its protecting role against self-damage from complement, and the FHRs are emerging as FH antagonists that act as an additional regulatory mechanism to control where and when FH protects human cells and/or surfaces. With the recent development of FHR-specific assays, quantification of the whole protein family has now become possible. This has elucidated the intricate balance between FH and the FHR proteins, showing that overall the balance is in favor of FH. However, this balance can shift on altered self, and also genetic variations have a major impact on FH and FHRs. This includes decreased FH function due to mutations, altered expression levels, as well as hybrid FH::FHR and FHR::FHR proteins and unusual FHR multimers with abnormal function that disturb complement regulation. Associations of increased FHR levels, as a result of genetic variations, with diseases like aHUS and IgAN are highly suggestive of a pathological role for the FHRs. It remains to be seen whether the FHRs are indeed causative in these diseases, but it is likely that they at least contribute to altered complement regulation on host surfaces.

## Author Contributions

PS-C, RBP, ML-T, and MJ prepared the text and the figures. All authors have revised and approved the manuscript.

## Conflict of Interest Statement

RBP is co-inventor of a patent describing potentiating anti-FH antibodies and uses thereof. The other authors declare no conflict of interest.
